# Convective influx/glymphatic system: tracers injected into the CSF enter and leave the brain along separate periarterial basement membrane pathways

**DOI:** 10.1007/s00401-018-1862-7

**Published:** 2018-05-12

**Authors:** Nazira J. Albargothy, David A. Johnston, Matthew MacGregor-Sharp, Roy O. Weller, Ajay Verma, Cheryl A. Hawkes, Roxana O. Carare

**Affiliations:** 1 Faculty of Medicine, University of Southampton, Southampton, UK; 2 Biogen, Cambridge, USA; 3 Open University, Milton Keynes, UK

**Keywords:** Intramural periarterial drainage, Basement membranes, Interstitial fluid, Cerebrospinal fluid, Glymphatic

## Abstract

Tracers injected into CSF pass into the brain alongside arteries and out again. This has been recently termed the “glymphatic system” that proposes tracers enter the brain along periarterial “spaces” and leave the brain along the walls of veins. The object of the present study is to test the hypothesis that: (1) tracers from the CSF enter the cerebral cortex along pial-glial basement membranes as there are no perivascular “spaces” around cortical arteries, (2) tracers leave the brain along smooth muscle cell basement membranes that form the Intramural Peri-Arterial Drainage (IPAD) pathways for the elimination of interstitial fluid and solutes from the brain. 2 μL of 100 μM soluble, fluorescent fixable amyloid β (Aβ) were injected into the CSF of the cisterna magna of 6–10 and 24–30 month-old male mice and their brains were examined 5 and 30 min later. At 5 min, immunocytochemistry and confocal microscopy revealed Aβ on the outer aspects of cortical arteries colocalized with α-2 laminin in the pial-glial basement membranes. At 30 min, Aβ was colocalised with collagen IV in smooth muscle cell basement membranes in the walls of cortical arteries corresponding to the IPAD pathways. No evidence for drainage along the walls of veins was found. Measurements of the depth of penetration of tracer were taken from 11 regions of the brain. Maximum depths of penetration of tracer into the brain were achieved in the pons and caudoputamen. Conclusions drawn from the present study are that tracers injected into the CSF enter and leave the brain along separate periarterial basement membrane pathways. The exit route is along IPAD pathways in which Aβ accumulates in cerebral amyloid angiopathy (CAA) in Alzheimer’s disease. Results from this study suggest that CSF may be a suitable route for delivery of therapies for neurological diseases, including CAA.

**Electronic supplementary material:**

The online version of this article (10.1007/s00401-018-1862-7) contains supplementary material, which is available to authorized users.

## Introduction

Apart from blood, there are two major extracellular fluids associated with the brain and spinal cord, namely CSF in the ventricles and subarachnoid spaces, and interstitial fluid (ISF) in the extracellular spaces of the brain and spinal cord. CSF and ISF appear to drain from the brain along largely separate pathways to regional lymph nodes [11]. Tracers injected into the CSF drain from the subarachnoid space to cervical lymph nodes by channels that pass through the cribriform plate and join lymphatic channels in the nasal submucosa [18, 20, 27]. Similarly, there is lymphatic drainage of CSF alongside cranial and spinal nerves and into lymphatics in the dura mater [3, 8, 11, 22].

There are no conventional lymphatic vessels in the brain parenchyma, but there is lymphatic drainage of ISF to cervical lymph nodes [11, 39]. When minute amounts of radioactive tracer are injected into deep grey matter of the rat brain, they drain to cervical lymph nodes along the walls of cerebral and intracranial arteries [39]. High-resolution studies using formalin-fixable fluorescent tracers suggest that the drainage pathways for ISF and solutes from the brain are the basement membranes in the walls of cerebral capillaries and arteries [4]. Injection of fluorescent tracer in small amounts of 0.5 µL through fine 50 μm glass cannulae ensures that no tracer leaks into the CSF [4]. At 5 min following these injections, tracers are within the basement membranes of cerebral capillaries and in basement membranes around smooth muscle cells in the tunica media of cerebral arteries. By 30 min post-injection, no tracer remains in basement membranes in the walls of capillaries or arteries, but the course of each artery involved is outlined by perivascular macrophages containing tracer [4].

Flow of tracer along smooth muscle cell basement membranes in the walls of arteries has been demonstrated by in vivo multiphoton imaging [2]. Ultrastructural studies have shown that, following injection of soluble amyloid-β (Aβ) into the mouse brain as a tracer, Aβ pervades the extracellular spaces of the brain and is in continuity with Aβ within capillary basement membranes [24]. In artery walls, however, tracer injected into the brain parenchyma is confined to basement membranes surrounding smooth muscle cells in the tunica media and is not observed in basement membranes associated with either endothelial cells or with pial-glial basement membranes on the external aspects of the tunica media [4]. It appears, therefore, that tracer is unlikely to enter artery walls either via the endothelium or radially from the brain parenchyma through the glia limitans. From these observations, it appears that tracers in the ISF pass from the brain parenchyma into basement membranes of capillaries and flow out of the brain along smooth muscle cell basement membranes in the walls of arteries [2, 4]. This pathway has been termed the intramural peri-arterial drainage (IPAD) pathway. The relevance of IPAD to neurodegenerative disease in humans is emphasized by the pattern of deposition of Aβ in the walls of cerebral capillaries and arteries as cerebral amyloid angiopathy (CAA). In the early stages of CAA, the pattern of deposition of Aβ corresponds, exactly, with the IPAD pathways identified by fluorescent tracer studies in the mouse [14, 19, 29].

Although tracers and Aβ in CAA outline the endothelial/glial basement membranes around capillaries, tracers and Aβ are limited to basement membranes around smooth muscle cells in the tunica media of arteries within the brain and leptomeninges. Neither the endothelial basement membranes nor the outer pial-glial basement membranes are outlined by tracer or by the deposition of Aβ [4].

There is, therefore, evidence from experimental studies and from CAA that IPAD is a physiological pathway for drainage of ISF and solutes from the brain. Evidence from radioactive tracer studies [36] and from the drainage of Aβ from the brain [26] strongly support the concept that the IPAD route is the lymphatic drainage pathway for ISF and solutes from rodent and human brain. Radioactive tracers injected into the brain are present in the walls of intracranial arteries but not in the carotid artery in the neck [36]. Furthermore, biochemical analyses of Aβ in the walls of human arteries from 20 years of age onwards detected increasing amounts of Aβ in the walls of intracranial arteries with age but very little Aβ was detected at any age in the extracranial carotid arteries in the neck [33]. These results would correlate with Aβ draining from the brain along artery walls and passing to lymph nodes closely applied to the carotid artery below the base of the skull [7].

Despite the largely separate drainage pathways for CSF and ISF, the two fluids do mix within the brain. Tracers injected into the CSF pass into the brain and spinal cord tissue along the outer aspects of arteries and this system has been variously named as convective tracer influx and the glymphatic system [17, 30]. Electron microscope studies have shown that there are no perivascular spaces around arteries in the cerebral cortex [23, 37, 40] although dilated perivascular spaces do develop in other parts of the brain such as cerebral white matter and the basal ganglia [32]. Perivascular spaces in the basal ganglia have been demonstrated by electron microscopy [28].

When nanoparticles are injected as tracers into the CSF of mice, they appear to pass into the brain along the basement membrane between the coatings of pia mater on the outer aspect of cortical arteries and the glia limitans on the surface of the brain, the pial-glial basement membrane [24]. Previous studies have suggested that tracers injected into the CSF pass into the brain along assumed periarterial “spaces” and drain out of the brain along the walls of veins [17]. Having defined by electron microscopy the most probable entry pathway for particulate tracers passing into the brain from the CSF as the pial-glial basement membrane, we used injections of tracer into the CSF of the cisterna magna of mice to achieve three objectives. Our first two major objectives in the present study are: (1) to demonstrate by immunocytochemistry that the pial-glial basement membranes are the entry route into the brain for soluble tracers from the CSF and (2) to test the hypothesis that tracers entering the brain from the CSF drain out of the brain parenchyma along the IPAD pathways. Groups of mice were examined 5 min and 30 min after injection of fluorescent Aβ tracer into the cisterna magna of young 2–10-week- and aged 24–30-month-old mice.

The third major objective in this study is to determine the range of depths of penetration of tracer alongside arteries in different regions of the brain and for the time points of 5 and 30 min in young and aged mice. The importance of this study relates to drug delivery from the CSF and will be dealt with further in the discussion.

## Materials and methods

### Animals

Male C57BL/6 mice 6–10 weeks (young) and 24–30 months (old) were housed at the Biomedical Research Facility (BRF) at Southampton General Hospital (Southampton, UK). All mice were housed in groups of 4–10. They were kept under standard 12-h light/dark cycle and fed a standard RM1 chow diet (SDS, UK) and water ad libitum. Environmental enrichment consisted of wooden sticks, red plastic tunnels and other bedding. All procedures were carried out in accordance with animal care guidelines stipulated by the United Kingdom Animals (Scientific Procedures) Act 1986, Home Office licence (PPL 30/3095).

### Anaesthesia

C57BL/6 mice were anaesthetised using a standard isoflurane anaesthesia system. An anaesthesia induction chamber was precharged with 5% isoflurane for 5 min and the oxygen flow meter was set to approximately 1.7 l min^−1^ with the indicator ball floating between the 1 and 2 hash marks. The isoflurane vaporizer was then reduced to 1–2% and the mouse was left in the chamber for 3–5 min. We chose isoflurane after our pilot experiments in *n* = 3 wild type mice determined that isoflurane did not reduce the heart rate or oxygen saturation in C57BL/6 mice to the same degree as our previous anaesthetic (ketamine and xylazine), see Online Resource 1. The level of anaesthesia was monitored using the pedal withdrawal reflex response. Once the mouse reached a surgical plane of anaesthesia (no reflex), it was transferred from the induction chamber onto a surgical bed with a breathing device and mask that supplied a constant flow of 1–2% isoflurane and 1.7 l min^−1^ oxygen to keep the animal safely anesthetised during the surgical procedure. The core body temperature was monitored using a rectal probe and maintained at 37 °C using an electronic heating pad. The cornea was protected using Lacri-Lube eye ointment.

### Intracisternal injections

The mouse was placed prone on a stereotaxic frame and head-restrained with ear bars after the area between the head and shoulders was shaved. Under a dissection microscope, a 10 mm midsagittal incision of the skin was made inferior to the occipital crest. To expose the dura covering the cisterna magna, the subcutaneous tissue and occipital muscles (biventer cervicis and rectus capitis dorsalis) were separated using blunt-ended forceps and the posterior atlanto-occipital membrane was incised. Using a cotton swab, the area was treated with a cotton bud impregnated with viscous glycerol to help prevent CSF reflux. A glass capillary micropipette (Sigma, UK) with an adjusted diameter (of < 50 μm, made using a Sutter P97 Flaming Brown Pipette puller) was positioned perpendicular to the ear bars and advanced gently to penetrate the dura until resistance was overcome indicating entry into the cisterna magna. The capillary had been loaded with 2 μl of 100 μM Aβ1-40 Hylite Fluor 555 (Bioscience), which was injected over 2.5 min at a rate of 0.8 μl min^−1^. The microcapillary was left in situ for 2 min to prevent reflux and mice were culled 5 or 30 min after withdrawal of the glass capillary through overdose with pentobarbital (200 mg kg^−1^). Mice were intracardially perfused with 0.01 M phosphate buffer saline (PBS) followed by 4% paraformaldehyde (PFA) in 0.01 M PBS at a rate of 5 ml min^−1^. Brains were dissected from the skull, post fixed in 4% PFA for 6–7 h and cryoprotected with 30% sucrose in 0.01 M PBS for 48 h. The brains were sectioned into 20-μm-thick coronal slices using a cryostat and stored at − 20 °C.

### Double-labeling immunofluorescence

For double-labelling immunohistochemistry, tissue sections were incubated overnight with anti-α-smooth muscle actin (SMA) FITC (1:200, Sigma-Aldrich, Dorset, UK) for the identification of arteries. Anti-collagen IV (1:400, ABCAM, Cambridge, UK) was used to mark the basement membranes of blood vessels (Thermo Fisher Scientific, Paisley, UK). Brain sections were also incubated with rat monoclonal anti-α-2 laminin (1:200) to identify the astrocyte basement membranes of the glia limitans abutting the outer aspect of cortical arteries [13] or mouse monoclonal anti-GFAP (1:100) or polyclonal rabbit anti-CD163 (1:100) at 4 °C for 24 h in a moist chamber. All primary antibodies were diluted in 0.1% PBST. Subsequently, sections were incubated with the appropriate secondary antibodies conjugated to Alexafluor fluorophores. Photomicrographs of triple labelling immunohistochemistry were captured across the brain starting anterior from the olfactory bulbs moving posteriorly towards the cerebellum. Sections were analyzed using an SP8 confocal laser-scanning microscope (Milton Keys, UK) and exported to Image J software. Montages were created with Photoshop CS6.

### Cerebral blood vessel classification

Arteries were differentiated from veins using an SMA marker, which only stains the smooth muscle cells that are present in arteries and arterioles. Vessels that were negative for SMA and had a diameter < 10 μm were classified as capillaries.

### Quantitative assessment of Aβ-positive blood vessels

To compare the depth of tracer 5 min and 30 min post-intracerebral injection in young and old mice, the distance of the Aβ-positive blood arteries from the surface or base of the brain was measured by drawing two perpendicular lines (one travelling in the direction of the vessel and a tangent of the surface/base of the brain; see Online Resource 2) and determining the distance of the vessel from the point at which the two lines meet. A total of 108 full coronal sections were analyzed in 6–10 week old mice (*n* = 3 per time point) and 56 sections in 24–30 month old mice (*n* = 3 per time point) and all the Aβ-positive blood SMA-positive arteries were quantified in this way. The sections quantified were taken at the level of the olfactory bulbs, cortex, ventricles, hippocampus, midbrain and cerebellum. The total length of each region (e.g., olfactory bulbs) was 180 μm.

### Statistical analysis

The average of all distances in each brain region per mouse were used for statistics (*n* = 3/group). Data were checked for normality using the D’Agostino & Pearson omnibus and Shapiro–Wilk normality tests. As the data in some groups were not normally distributed, all values were Log_10_ transformed to obtain a normal distribution. One-way analysis of variance (ANOVA) with Sidak’s post hoc test was used to compare the distance at which Aβ was seen from the reference point at the surface of the brain within the same brain region at 5 and 30 min after intracisternal injections. Two-way ANOVA with Sidak’s post hoc test was used to compare the distance at which Aβ was seen from the reference point at the surface of the brain between young and old mice at 5 and 30 min after intracisternal injections. Statistical analyses were performed using log-transformed data, with corresponding p values indicated on graphs that were generated using non-transformed data.

## Results

### Anatomical distribution of β-amyloid after its injection into cisternal CSF

First, the possible route of entry of Aβ1-40 (referred to from here as Aβ) contained within the CSF into the brain of 6–10-week old mice was evaluated by double-labelling immunohistochemistry and confocal microscopy. The anatomical distribution of Aβ in the brain and the type of Aβ-positive blood vessels were determined by staining the sections with an SMA marker to differentiate arteries from veins, a general basement membrane marker (collagen IV) and a specific astrocytic basement membrane marker (α-2 laminin). The anatomical compartment in which the tracer was contained was defined and compared after 5 and 30 min of injection of tracer into the cisterna magna.

#### 6–10-week-old mice, 5 min post-injection

**Fig. 1:**
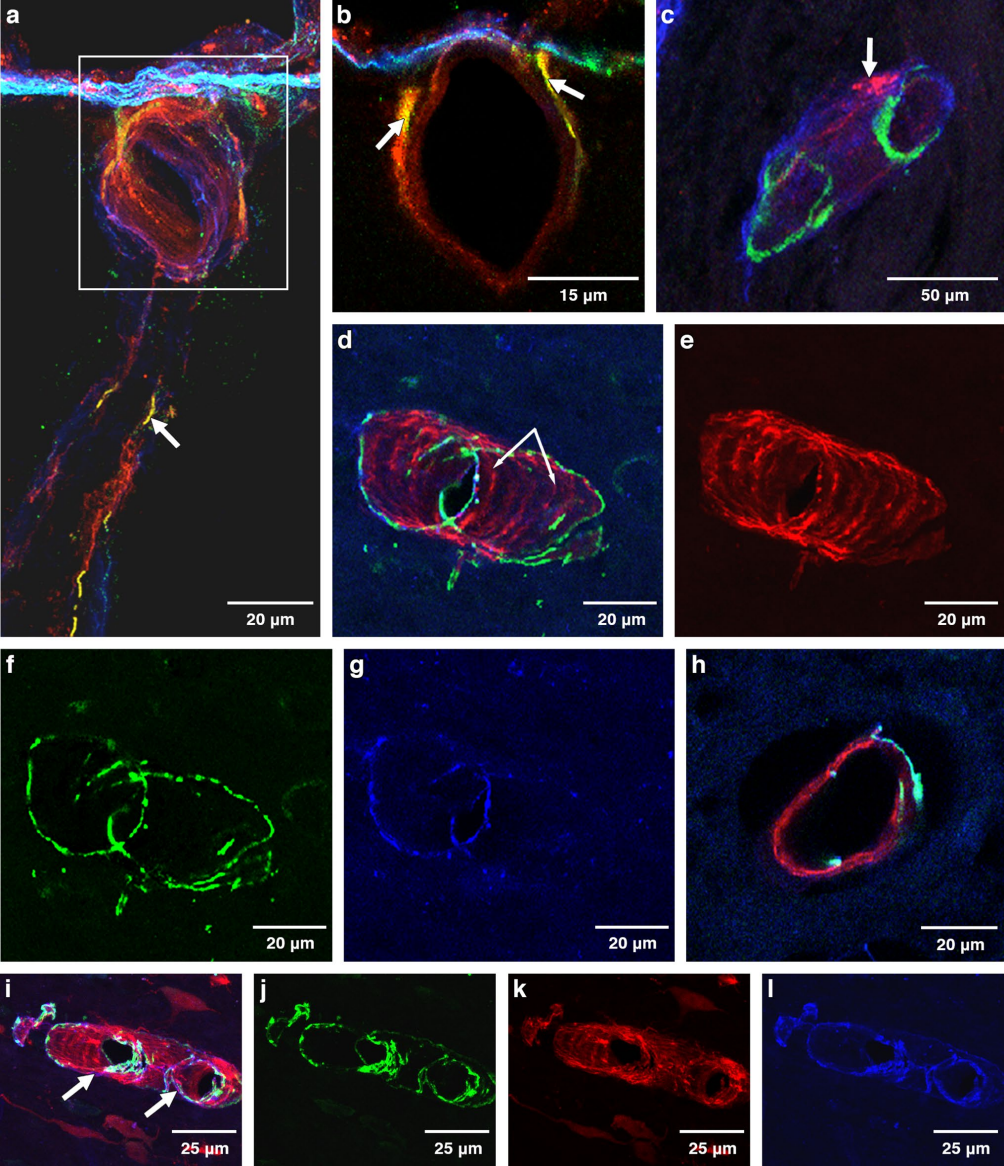
Anatomical entry route of Aβ into the young adult brain from the CSF at 5 and 30 min post-injection. Young 6–10-week-old mice: Entry of amyloid-β (Aβ) tracer into the brain from the CSF at 5 min (**a**–**c**). Drainage of Aβ tracer from the brain along the walls of arteries at 30 min (**d**–**l**). **a** 5 min: the surface of the cerebral cortex (blue line) and a cortical artery passing into the brain with Aβ tracer red in its walls. Aβ tracer colocalized with α2-laminin in the pial-glial basement membrane (yellow) indicated by the white arrow. **b** 5 min: a single optical section of the artery enclosed in the box in **a** showing α2-laminin in the astrocyte basement membrane of the glia limitans on the surface of the brain (green) and co-localisation of Aβ and α2-laminin in the pial-glial basement membrane on the outer aspect of the wall of the artery (yellow) (white arrow); **c** 5 min: the profile of a cortical artery with smooth muscle cells (green) in its wall shows Aβ tracer (red) colocalized (pink) with collagen IV (blue) in the pial basement membrane on the outer surface of the artery (white arrows). At 30 min after injection as shown in figures (**d**–**l**), the distribution of the Aβ in the artery walls is very different from that seen at 5 min in figures (**a**–**c**). **d** An artery identified by green smooth muscle actin in its wall (**f**, **j**) shows Aβ tracer (red, **e**, **k**) colocalized (pink) with collagen IV (blue, **g**, **l**) within the wall of the artery in a spiral or ladder-type distribution (arrows in **d**), closely resembling the pattern of deposition of Aβ in the walls of arteries as cerebral amyloid angiopathy (CAA); **h** a single optical section from the artery in **d**) showing the Aβ tracer (red) between the smooth muscle actin staining (green)

Aβ was detected within the walls of leptomeningeal and cortical arteries (Fig. 1). Immunocytochemistry with multiple labelling of collagen IV, α-2 laminin and Aβ tracer confirms the presence of Aβ tracer in the brain-disposed along pial-glial basement membranes on the outer aspects of arteries. Figure 1a shows a blood vessel penetrating the cerebral cortex with Aβ tracer, stained red, in the vessel wall. Co-localisation of Aβ tracer and α-2 laminin in the pial-glial basement membrane is shown as yellow. Co-localisation of Aβ tracer and collagen IV in the artery wall is depicted as fuchsia pink. At higher magnification in Fig. 1b, α-2 laminin in the astrocyte basement membranes of the glia limitans on the surface of the brain is stained green and co-localisation of Aβ and α-2 laminin wall of the artery is shown as yellow. Confirmation that the Aβ tracer is in basement membranes on the outer aspect of arteries 5 min after injection into the CSF is seen in Fig. 1c. The blood vessel in longitudinal view is identified as an artery by the presence of smooth muscle cells stained green for smooth muscle actin in its wall. Aβ tracer co-localizes with collagen IV (blue) as a fuchsia pink colour in a single fine layer of basement membrane on the outer aspects of the artery. This corresponds to the position on the outer aspect of the artery to the pial-glial basement membrane [24].

#### 6–10-week-old mice, 30-min post injection

Aβ was detected in the basement membranes surrounding smooth muscle cells within the walls of leptomeningeal and cortical arteries but not in the walls of capillaries and veins. The vessel in longitudinal view in Fig. 1d is an artery identified by the smooth muscle cells in its wall stained green by α smooth muscle cell actin (Figs. 1d, f, j). At 30 min after injection into the cisterna magna, Aβ tracer (Fig. 1e, k) 1 is seen in a spiral configuration that represents the smooth muscle cell basement membranes within the artery wall; the fuchsia pink staining (Fig. 1d, i) represents co-localisation of Aβ tracer in the walls of arteries in Fig. 1e, k and collagen IV in the smooth muscle cell basement membranes (Fig. 1g, j, l). In a single optical section from an arteriole, fuchsia pink colocalisation of collagen IV and Aβ is seen running between green-stained smooth muscle cells in the tunica media of the arteriole (Fig. 1h).

#### 24–30 month old mice, 5-min post injection

**Fig. 2:**
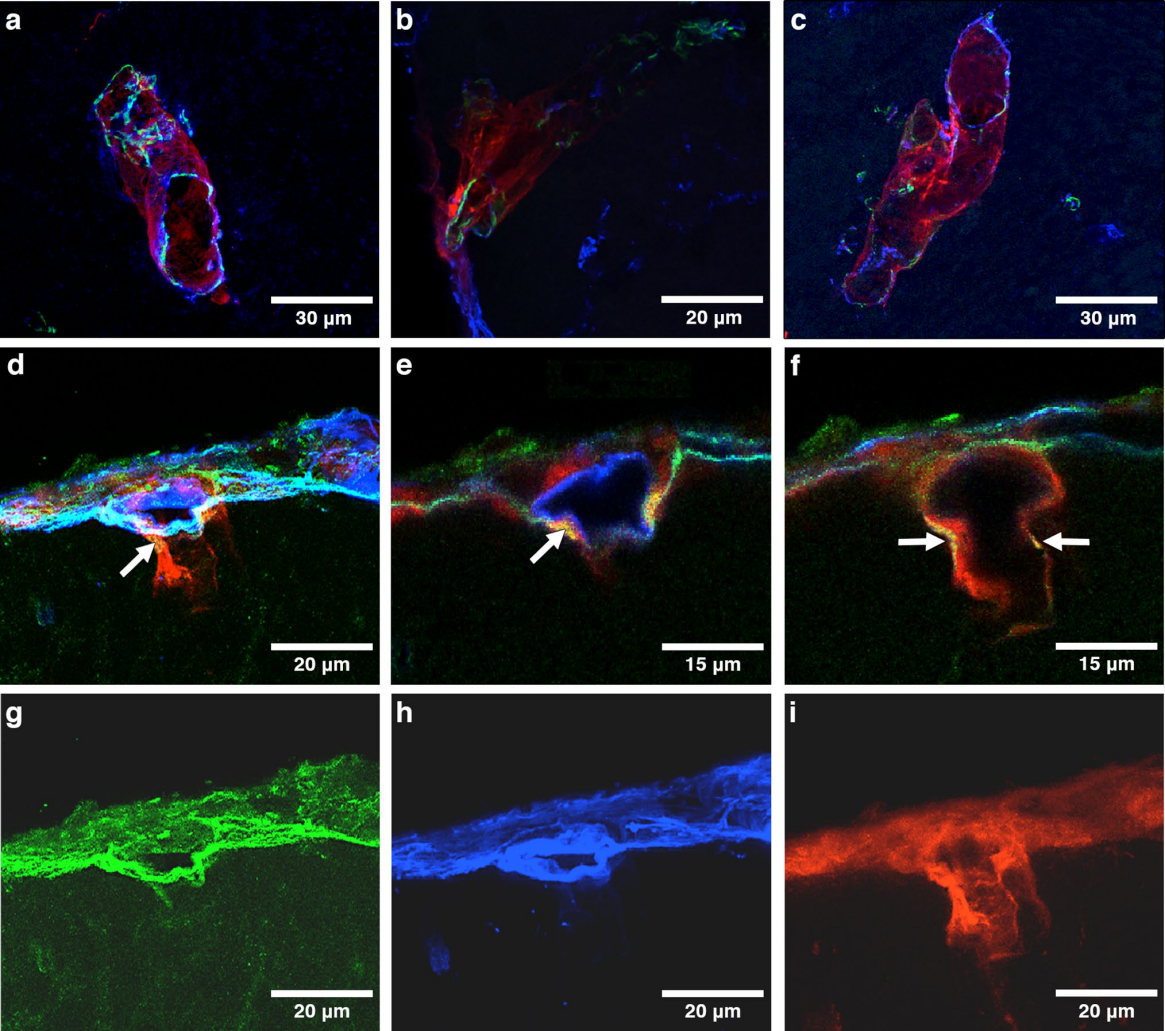
Anatomical entry route of Aβ into old 24–30-month-old mouse brain from the CSF at 5 min post injection: **a**–**c** arteries showing Aβ (red) along the walls of arterioles of 10–20 µm diameter labelled with collagen IV (blue) and smooth muscle actin (green); **d** an artery entering the cerebral cortex (arrow) showing Aβ (red) extending into the brain; **e** enlargement of the artery in (**d**) showing laminin α-2 staining in the glia limitans on the surface of the brain (green) and co-localisation of the amyloid with laminin α-2 in the vessel wall (yellow) as indicated by the arrow; **f** single optical section of artery in **e**; **g** single channel image of laminin α-2 from **d**; **h** single channel image of collagen IV from **d**; **i** single channel image of Aβ from **d**

At 5 min following injection of Aβ tracer into the cisterna magna, the distribution of tracer is similar in the 24–30-month-old mice to that found in the young 6–10-week-old mice. Aβ tracer was observed in the walls of arterioles, as depicted by immunostaining for green smooth muscle actin in Figs. 2a, and b and in the walls of capillaries, as shown in Fig. 2c. In Fig. 2d, a blood vessel penetrating the surface of the cerebral cortex shows Aβ tracer within the vessel wall and co-localizing with α-2 laminin indicating its presence in the pial-glial basement membrane on the abluminal aspect of the vessel. At higher magnification in a single channel image in Fig. 2e, and f, α-2 laminin in the glia limitans on the surface of the brain is stained green. Co-localisation of α-2 laminin and Aβ tracer in the pial-glial basement membrane on the outer aspect of the vessel wall is depicted in yellow.

#### 24–30-month-old mice, 30 min post injection

**Fig. 3:**
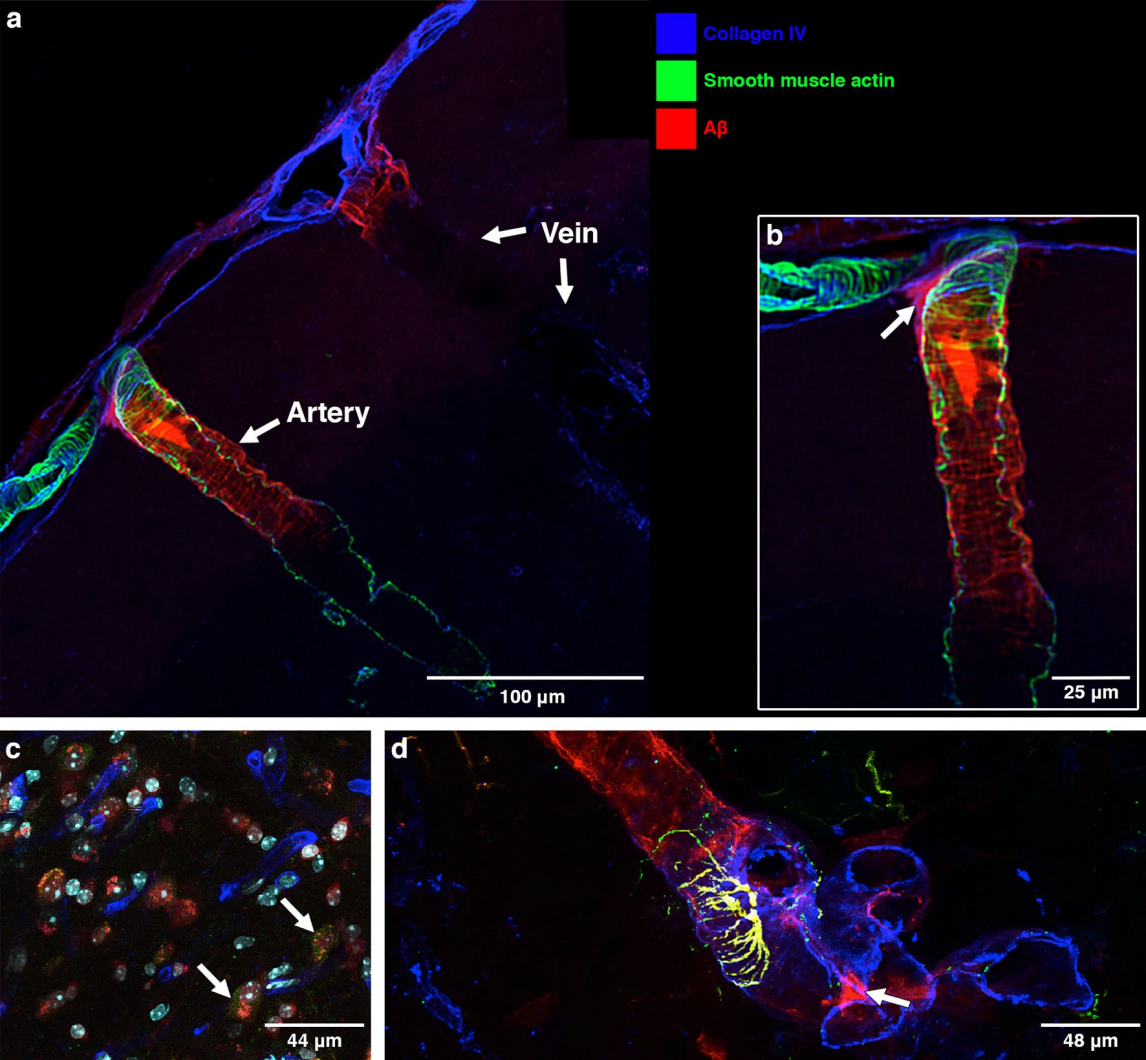
Anatomical route for drainage of Aβ out of the brain following entry from the CSF at 30 min post injection in old 24–30-month-old mice: **a** An artery and vein at the surface of the brain. The artery is identified by smooth muscle cells in its wall (green). Aβ tracer (red) is present in a ladder-like pattern in the wall of the artery. Aβ (red) in the wall of a vein is only seen at the surface suggesting that the Aβ here has entered from the CSF and not drained from the brain. Only occasional veins had Aβ in their walls. **b** Enlargement of the artery in **a** showing the ladder-like distribution of Aβ that resembles the distribution of amyloid in CAA [29]. At the surface of the brain is a branch of a leptomeningeal artery showing the ladder-like distribution of smooth muscle cells in the tunica media. The Aβ is in the intramural periarterial drainage (IPAD) pathway; **c** macrophages take up Aβ tracer at 30 min after injection. The arrows indicate Aβ (red) within macrophages stained for the macrophage marker CD163 (green); **d** astrocytes are stained for GFAP (green). Co-localisation (yellow) of Aβ and GFAP indicates uptake of Aβ tracer by perivascular astrocytes

The pattern of distribution of Aβ tracer in the walls of blood vessels in the older mice at 30 min after intracisternal injection is slightly different from that in the young mice. With regard to the arteries, the distribution of Aβ tracer is the same in the two age groups. Figure 3a shows an artery and vein at the surface of the cerebral cortex. The artery is identified by the staining of smooth muscle cells in its wall; Aβ is present in the same spiral pattern as smooth muscle cell basement membranes in the tunica media. A leptomeningeal artery in Fig. 3a, b shows the corresponding spiral pattern of smooth muscle cells in the tunica media. Aβ tracer is present in a short region of the wall of a vein near the subarachnoid space in Fig. 3a. Aβ tracer, red, colocalizes with cells stained green for the macrophage marker CD 163 (Fig. 3c). In addition, Aβ tracer co-localizes with glial fibrillary acidic protein (GFAP) in perivascular astrocyte processes in Fig. 3d.

### Periarterial penetration of β-amyloid tracer into the brain parenchyma

Next, the periarterial distances of Aβ from the surface of the brain at 5 and 30 min after intracisternal injection were determined using the quantification method illustrated in Online resource 1. Normally, distributed data presented in the form of histograms show the Log_10_ values.

### Regional differences in distance of periarterial penetration of Aβ into the parenchyma after 5 min in 6–10-week-old mice

Soluble Aβ injected into the CSF was only observed along the walls of leptomeningeal and cortical arteries after 5 and 30 min of injection. No Aβ was observed in the walls of capillaries or veins. The depth of penetration of Aβ along the walls of cortical arteries was calculated in eleven different brain regions. At 5 min post injection, there was no statistically significant difference between the periarterial Aβ distance in the olfactory bulbs and the frontal, parietal, and entorhinal cortices. However, the distance at which Aβ was detected in the walls of arteries was significantly greater in the visual cortex, caudoputamen, thalamus, pons and cerebellar molecular layer compared to the olfactory bulbs (*P* < 0.0001). The greatest distance at which Aβ was detected from the surface of the brain was 253.18 µm and this was observed in the pons. No Aβ was detected in the corpus callosum.

### Regional differences in the distance of periarterial penetration of Aβ after 30 min in 6–10-week-old mice

**Fig. 4:**
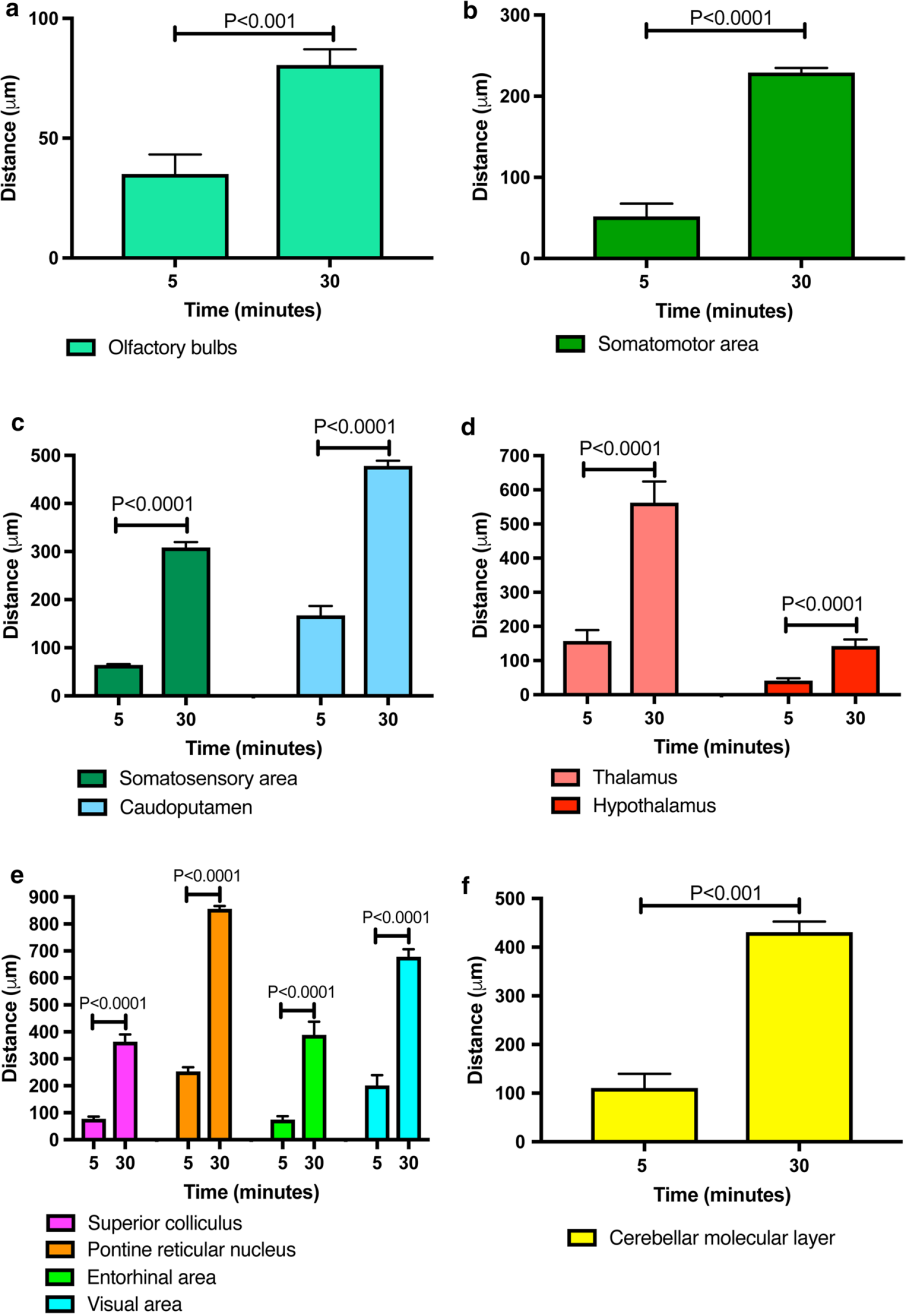
Periarterial penetration of Aβ is of greater distance after 30 min than after 5 min in cortical, subcortical and posterior brain regions of young mice. Bar charts of periarterial Aβ distance against time after injection into cisternal CSF (5 and 30 min) in the olfactory bulbs (**a**), somatomotor area (**b**), somatosensory area and caudoputamen (**c**), thalamus and hypothalamus (**d**), superior colliculus, pontine reticular nucleus, entorhinal and visual areas (**e**) and cerebellar molecular layer (**f**). Values are presented as mean ± SEM of untransformed data, with p values indicated for log10-transformed data (one-way ANOVA with Sidak’s post hoc)

Regional differences in the depth from the brain surface at which Aβ within the walls of arteries was located in the parenchyma at 30 min from the intracisternal injection, were largely similar to those detected after 5 min. The main differences were in greater depths of penetration in the olfactory bulbs and the cortical, subcortical and posterior brain regions. There was a statistically significant difference in the distance at which periarterial Aβ was detected within the parenchymal arteries in all eleven regions compared to the olfactory bulbs (*P* ≤ 0.0001), Fig. 4.

### Regional differences in the periarterial distance of Aβ penetration into the parenchyma in 24–30-month-old mice

After 5 min of injection into the cisterna magna of 24–30-month-old mice, the greatest periarterial distance along which Aβ was detected was in the pons and the least was detected in the thalamus. The periarterial Aβ distance in the olfactory bulbs was significantly greater than the frontal cortex and the thalamus, but significantly less than the caudoputamen, tectum/tegmentum and pons (*P* < 0.0001). The periarterial Aβ distance in the caudoputamen was significantly higher than in the frontal cortex, thalamus and tectum/tegmentum.

**Fig. 5:**
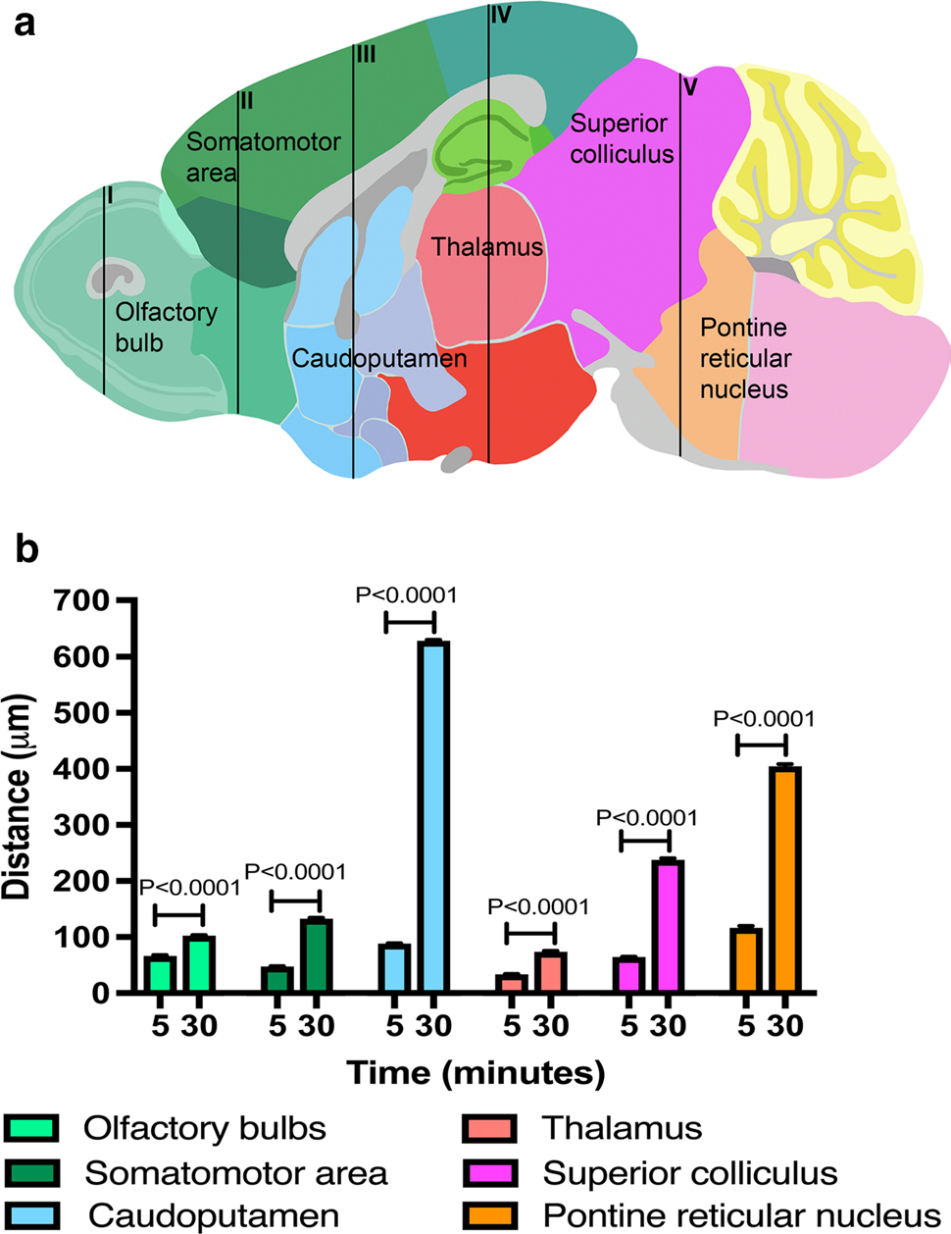
Regional differences in periarterial distance of penetration of Aβ at 5- and 30-min post-injection in old mice. **a** Schematic showing a sagittal mouse brain section with five brain levels (I–V) from which distance measurements were taken in six brain regions at 5 min after injection of Aβ into cisterna magna. (Allen Institute for Brain Science. Allen Mouse Brain Atlas. Available from: http://mouse.brain-map.org/static/atlas.) **b** Bar charts of periarterial Aβ distance against time after injection into cisternal CSF (5 and 30 min) in the olfactory bulbs (teal), somatomotor area (green), caudoputamen (blue), thalamus (salmon), superior colliculus (pink) and pontine reticular nucleus (orange). Values are presented as mean ± SEM of untransformed data, with p values indicated for log10-transformed data (one-way ANOVA with Sidak’s post hoc)

At 30 min, the greatest periarterial distance at which Aβ was observed in the parenchyma was no longer in the pons but in the caudoputamen, followed by the pons. Similar to the young mice, the periarterial distance of Aβ was significantly greater after 30 min compared to 5 min in all of the brain regions analysed including the olfactory bulbs, frontal cortex, caudoputamen, thalamus, tectum/tegmentum and pons (one-way ANOVA, *P* < 0.0001), Fig. 5.

The comparison between the patterns of the periarterial distance of Aβ entry from the CSF into the parenchyma at 5 and 30 min after intracisternal injection for both young and old mice is presented in Online Resource 3.

## Discussion

The first two major objectives in the present study were: (1) to demonstrate by immunocytochemistry that the pial-glial basement membranes are the entry route into the brain for soluble tracers from the CSF and (2) to test the hypothesis that tracers entering the brain from the CSF drain out of the brain parenchyma along the IPAD pathways that are lymphatic drainage pathways from the brain for the elimination of fluid and solutes.

To fulfil these objectives, we performed multi-labelling immunocytochemistry and confocal microscope studies on young 6–10-week-old mice and on aged 24–30-month-old mice, both at two separate time points of 5 and 30 min after injection of Aβ tracer into the CSF of the cisterna magna.

**Fig. 6:**
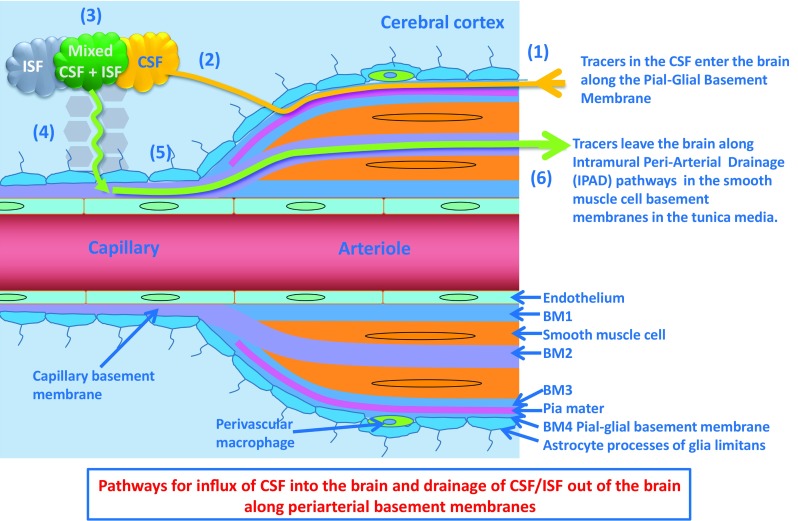
Pathways for influx of CSF into the brain and drainage of CSF/ISF out of the brain along capillary and periarterial basement membranes. (1) Entry of tracers from the CSF into the brain along pial-glial basement membranes on the outer aspects of artery walls. The sites of entry of tracer into the brain extracellular compartment are unclear and could be multiple. (2) CSF enters the brain parenchyma and (3) mixes with interstitial fluid (ISF). (4) The mixture of CSF/ISF diffuses through the narrow extracellular spaces of the brain to enter (5) basement membranes in the walls of capillaries to drain out of the brain along (6) basement membranes of smooth muscle cells in the tunica media in the walls of arterioles and arteries (IPAD pathway). Key to the microanatomy of the capillary and artery walls: *BM1* endothelial basement membrane. *BM2* smooth muscle cell basement membrane identified in this study by the presence of collagen IV. *BM3* basement membrane between pia mater (pink) and smooth muscle cell. *BM4* pia-glial basement membrane between the pia mater and the astrocytes of the glia limitans. In this study, BM4 is identified by the presence of α2-laminin [9, 13]. *Proposed route of IPAD* tracer is located in the capillary endothelial basement membrane (5) apparently entering from the brain parenchyma. In the artery wall, tracer is observed in the basement membranes between smooth muscle cells (light purple) but not in the basement membranes on the outer aspect of the artery wall or in the endothelial basement membrane of the artery wall (both dull blue) [4, 27]. The light green arrow indicates the proposed intramural peri-arterial drainage (IPAD) pathway for fluid and solutes out of the brain

Our study showed similar but not exactly identical results in mice in the two age groups. We confirmed by immunocytochemistry and confocal microscopy, our previous electron microscope studies that, within 5 min of injection into the CSF, tracer were present in the brain within basement membranes on the outer aspects of cortical artery walls. More exactly, the electron microscope study [24] and the present results using immunocytochemistry for α-2 laminin [9] identified tracer in pial-glial basement membranes. These results suggest that one pathway for entry of tracers into the brain from the CSF is along the pial-glial basement membranes between the layer of pia mater and astrocytes in the perivascular glia limitans (Fig. 6). This entry pathway was observed in the young and in aged mice.

Basement membranes and cell components surrounding cortical arteries are compacted so that there is no perivascular “space” for transport of tracers [23, 37]; transport appears to be along basement membranes as shown in the present study. This contradicts the statement in the glymphatic literature that there is transport of tracers into the brain along perivascular “spaces” [17]. These authors used low resolution two-photon microscopy which did not resolve the microanatomy of the perivascular compartment. Furthermore, the subarachnoid space appears to have been mistaken for perivascular space in the electron micrographs [17].

At 30 min following injection into the CSF of the cisterna magna, in the present study, Aβ tracer colocalised with collagen IV in basement membranes within the walls of arteries. These basement membranes are in the position of smooth muscle cell basement membranes in the tunica media of arteries [4, 14, 27, 29] and correspond to the intramural peri-arterial drainage (IPAD) pathways that are the proposed route of drainage of interstitial fluid and solutes from the brain.

The pattern of distribution of Aβ tracer in the artery wall at 30 min after injection into cisternal CSF was similar in the young 6–10-week- and the old 24–30-month-old mice. Aβ tracer was located in the spirally arranged smooth muscle cell basement membranes within the tunica media of arteries within the cerebral cortex. This is the same IPAD pathway identified previously by intracerebral injections of tracer in mice and the same IPAD pathway in which the Aβ is deposited in CAA [5, 19, 29]. Electron microscope studies of CAA have shown that fibrillary Aβ is initially deposited in the lamina densa that forms the junction zone between basement membranes of adjacent smooth muscle cells in the tunica media [12, 38].

With the increase in volume of deposited Aβ in CAA, the mass of Aβ separates the basement membranes of adjacent smooth muscle cells [5, 19]. Although adjacent basement membranes are fused at the lamina densa in a normal artery, it appears that the two parts of the basement membrane can be separated by the deposition of amyloid [12]. Nevertheless, co-localisation of tracer with laminin in previous studies [4] and collagen IV in the present study suggest that bulk flow of tracers, therefore, of ISF and other solutes, out of the brain is along basement membranes that form the IPAD pathways.

The results of the present investigation suggest a possible route for connection between the inflow of tracers from the CSF along pial-glial basement membranes and the outflow along smooth muscle cell basement membranes in the IPAD pathway. In the older 24-30-month-old mice, Aβ tracer injected into the CSF was located within astrocytes and in macrophages in the brain parenchyma at 30 min. This suggests that tracer had entered the extracellular, ISF compartment of the brain and been taken up by these cells. Futhermore, Aβ, tracer was located in the walls of brain capillaries associated with collagen IV in the capillary basement membranes. It seems most probable that the Aβ tracer drains out of the brain initially along basement membranes in the walls of cerebral capillaries and then along smooth muscle cell basement membranes in the walls of cerebral arterioles and arteries. This is effectively the same IPAD pathway identified for the outflow of tracers injected directly into the brain parenchyma [4].

Previous studies have suggested that tracers entering the brain from the CSF drain out of the brain along the walls of veins [17]. This suggestion is derived not from injection of tracers into the CSF but from intracerebral injections of tracer. Veins were not conclusively identified in these studies, and it is unclear whether the tracer was even present in the walls of blood vessels or in the surrounding brain tissue. In Iliff et al., [17] the veins were identified by the absence of NG2Ds-Red rather than immunocytochemistry for the absence of smooth muscle actin [17]. Recent studies demonstrate that NG2Ds-Red is not specific for identification of arteries, so it is possible that some of the vessels that were NG2-DsRed negative may indeed have been smooth muscle actin positive arterioles [15].

It is also likely that in this study [17], tracer leaked from the brain into the CSF during the intracerebral injections due to the use of a large 33G needle for injecting a large 1 µl volume of tracer. This would account for the appearance of tracer as oval patches resembling macrophages surrounding veins in the subarachnoid space. Furthermore, the time of examination of the brain was one hour rather than the much earlier time of 5 min at which it has been shown that tracers, injected intracerebrally, drain out of the brain along IPAD pathways [4]. In the present study, tracer was only rarely detected in the walls of veins for short distances very near the surface of the brain and this probably represented incursion of tracer from the subarachnoid space. For example, the vein in Fig. 3a shows a small amount of Aβ (red) in its wall near the surface of the brain but no Aβ in the wall further into the brain. This short ingress of tracer into the brain from the SAS along veins has been recorded in [17] and recent studies [1, 13, 27].

Other recent studies cast doubt upon the circulation of Aβ from the periarterial convective influx compartment to the perivenous compartment for clearance into the CSF [34, 35]. Our results support the convective influx of Aβ along arterial perivascular compartments in the pial-glial basement membranes, but we did not observe perivenous clearance into the CSF. This field of work is significant not only for the pathogenesis of CAA, but also for the distribution of drugs into the parenchyma after intrathecal delivery, as well as for the interpretation of biomarkers in the CSF.

The third objective of the present study was to determine the range of depths of penetration of tracer alongside arteries in different regions of the brain at the time points of 5 and 30 min in young and aged mice. We demonstrated that the pons is the region with the highest penetration of Aβ at both ages at 5 min after intracisternal injection. Recently, we showed in vivo that entry of contrast agent is most efficient in the midbrain of dogs and the pial-glial basement membranes were also thickest in the midbrain [10]. This suggests that the brainstem is an area most efficiently reached by therapeutic agents from the CSF, most likely also explaining the success of intracisternally administered antisense oligonucleotides in the treatment of spinal muscular atrophy (SMA) [6, 21]. SPINRAZA is an anti-sense oligonucleotide that alters the splicing of survival motor neuron (SMN) pre-messenger RNA to increase production of full-length normal SMN protein. Following a randomized, double-blind, sham-controlled study in infantile-onset SMA where SPINRAZA (www.spinraza.com) was administered intrathecally, the motor milestones improved significantly and the numbers of deaths decreased. Our results suggest that it is possible for solutes to reach the neurons in the brainstem within 5 min increasing thus the understanding of how anti-sense oligonucleotides that are in development for intrathecal administration in the treatment of neurological diseases may reach their targets [31].

Although in the present study, the depth at which Aβ was observed in the brain increased after 30 min, most was observed in the pial-glial basement membranes of arteries. This suggests that therapeutic agents would need to then diffuse from the pial-glial basement membranes towards their target, reducing the concentration and therapeutic efficiency. Only 10–15% of the intrathecally administered dose of proteins in cynomolgus monkeys was observed within the parenchyma at 2.5 h after injection of tracer into the CSF and by 24 h this decreased to 2% [25].

The exact dynamics of biomarkers in the CSF associated with neuropathological conditions remain unclear. Although with advancement of Alzheimer’s disease, there is a decrease in the amount of both Aβ1-40 and 1-42 in the CSF, there is a corresponding increase in total and phosphorylated forms of tau [16]. It is probable that the Aβ becomes entrapped in the IPAD pathways as CAA, and therefore, less Aβ is able to reach the CSF; this is in contrast to the more soluble tau that may be able to filter more readily along the chains of glycoproteins and proteoglycan components of the basement membranes surrounding smooth muscle cells.

In conclusion, we suggest that the present observations support the working hypothesis that tracers injected into the CSF pass into the brain along pial-glial basement membranes on the outer aspects of cortical arteries, enter the brain parenchyma and drain out of the brain along pericapillary and periarterial basement membranes that form the IPAD pathway for the drainage of ISF and solutes from the brain. In older mice, this pathway is impaired, as by 30 min after intracisternal injection, the tracer is observed in the capillary basement membranes, as well as in astrocytes and macrophages.

## Electronic supplementary material

Below is the link to the electronic supplementary material.

Male C57BL/6 mice 10 weeks old (n=3) were anaesthetised either with isoflurane (1% with 0.8 litre/min O^2^) or with 10mg/ml ketamine & 1mg/ml xylazine. The oxygen saturation, heart and breathing rates were recorded for 10 minutes using a non-invasive infrared thigh sensor attached to a MouseOx Plus Oximeter running premium software (STARR Life Sciences, Holliston, Ma, USA). The isoflurane maintained the heart rate and oxygen pressure at physiological levels when compared to ketamine and xylazine (TIFF 311 kb)

Method for measuring distance of periarterial Aβ penetration into the brain. (a) Sagittal view of mouse brain illustrating the locations (I–VI) at which representative slices were taken to analyse the penetration of Aβ in the brain from the CSF. (© 2018 Allen Institute for Brain Science. Allen Mouse Brain Atlas. Available from: http://mouse.brain-map.org/static/atlas.) (A, I-VI) Sections were taken from the level of the olfactory bulbs, cerebral cortex, lateral ventricles, anterior hippocampus, midbrain/hindbrain and cerebellum. The total length of each level analysed per mouse (n = 3/group) was 180 μm (double headed arrow). (b) Coronal view of the brain levels selected for analysis; mouse brains for this study were sectioned coronally. (c) Confocal micrographs corresponding with the boxes in B, III (1-3). (c, 1) Micrograph illustrating two perpendicular lines, a tangent to the midline fissure in the cerebral and another line in the direction of the Aβ-positive vessel. (c, 2) Same quantification method applied to the Aβ-positive vessel in the caudoputamen in relation to the lateral ventricle (asterisk). (c, 3) Distance of Aβ-positive vessel measured in the striatum in relation to the base of the brain. Scale bar: 150 μm (TIFF 10671 kb)

Distance of periarterial Aβ penetration into the brain in young and old mice at 5 and 30 mins post injection. Bar charts of periarterial Aβ distance against time after injection into cisternal CSF (5 and 30 minutes) in the olfactory bulbs (a), somatomotor area (b), caudoputamen (c), thalamus (d), superior colliculus (e) and pontine reticular nucleus (f) of young and old mice. Values are presented as mean ± SEM of untransformed data, with p values indicated for log10-transformed data (two-way ANOVA with Sidak’s post hoc) (TIFF 553 kb)
