# Supplementary figures and images for: Convective influx/glymphatic system: tracers injected into the CSF enter and leave the brain along separate periarterial basement membrane pathways
Source: Acta Neuropathol. 2018 May 12;136(1):139–52. doi: 10.1007/s00401-018-1862-7 (PMC6015107; doi:10.1007/s00401-018-1862-7)

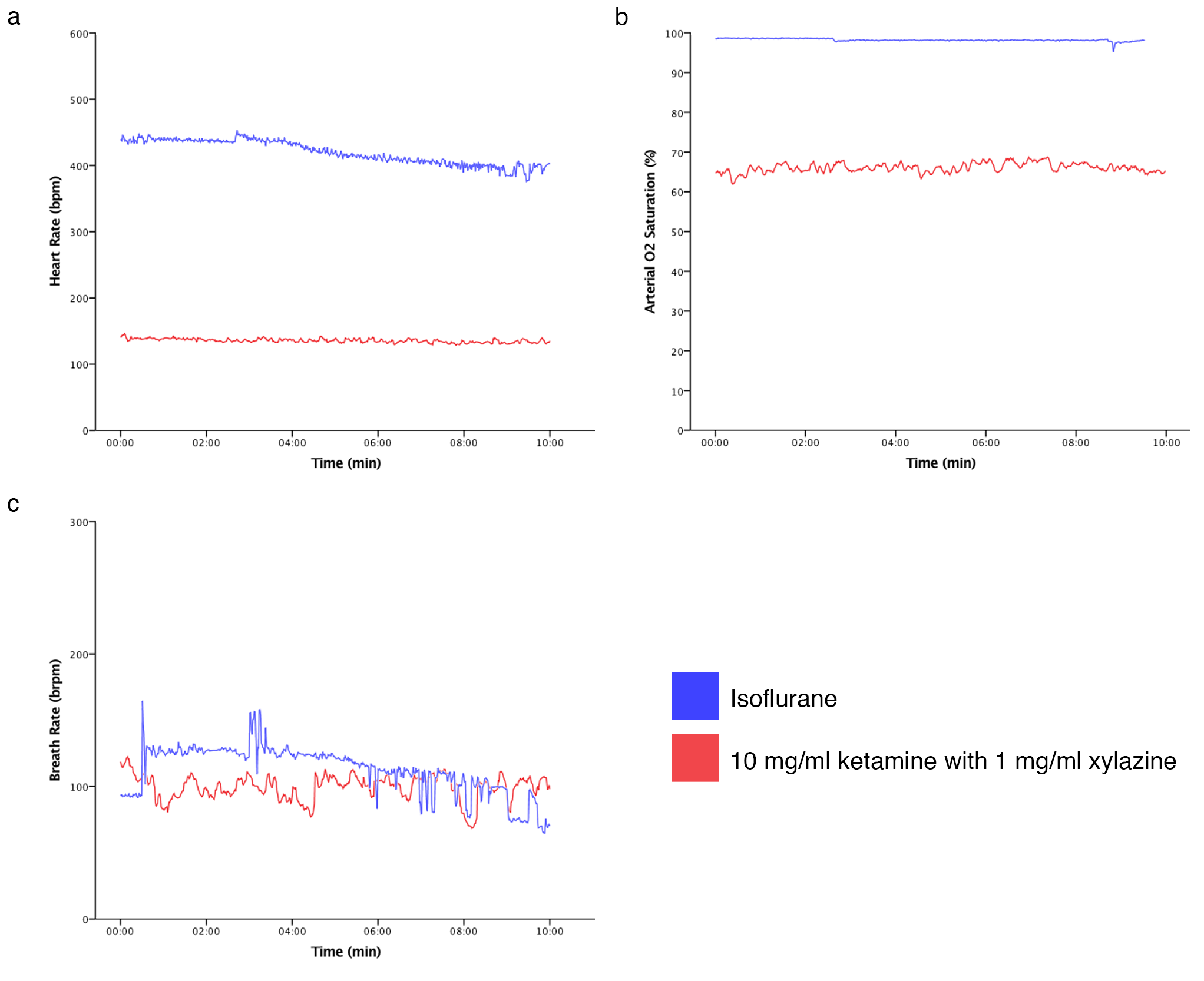

Supplement: Supplementary file 1 — Male C57BL/6 mice 10 weeks old (n=3) were anaesthetised either with isoflurane (1% with 0.8 litre/min O2) or with 10mg/ml ketamine & 1mg/ml xylazine. The oxygen saturation, heart and breathing rates were recorded for 10 minutes using a non-invasive infrared thigh sensor attached to a MouseOx Plus Oximeter running premium software (STARR Life Sciences, Holliston, Ma, USA). The isoflurane maintained the heart rate and oxygen pressure at physiological levels when compared to ketamine and xylazine (TIFF 311 kb) [file 401_2018_1862_MOESM1_ESM.tiff]

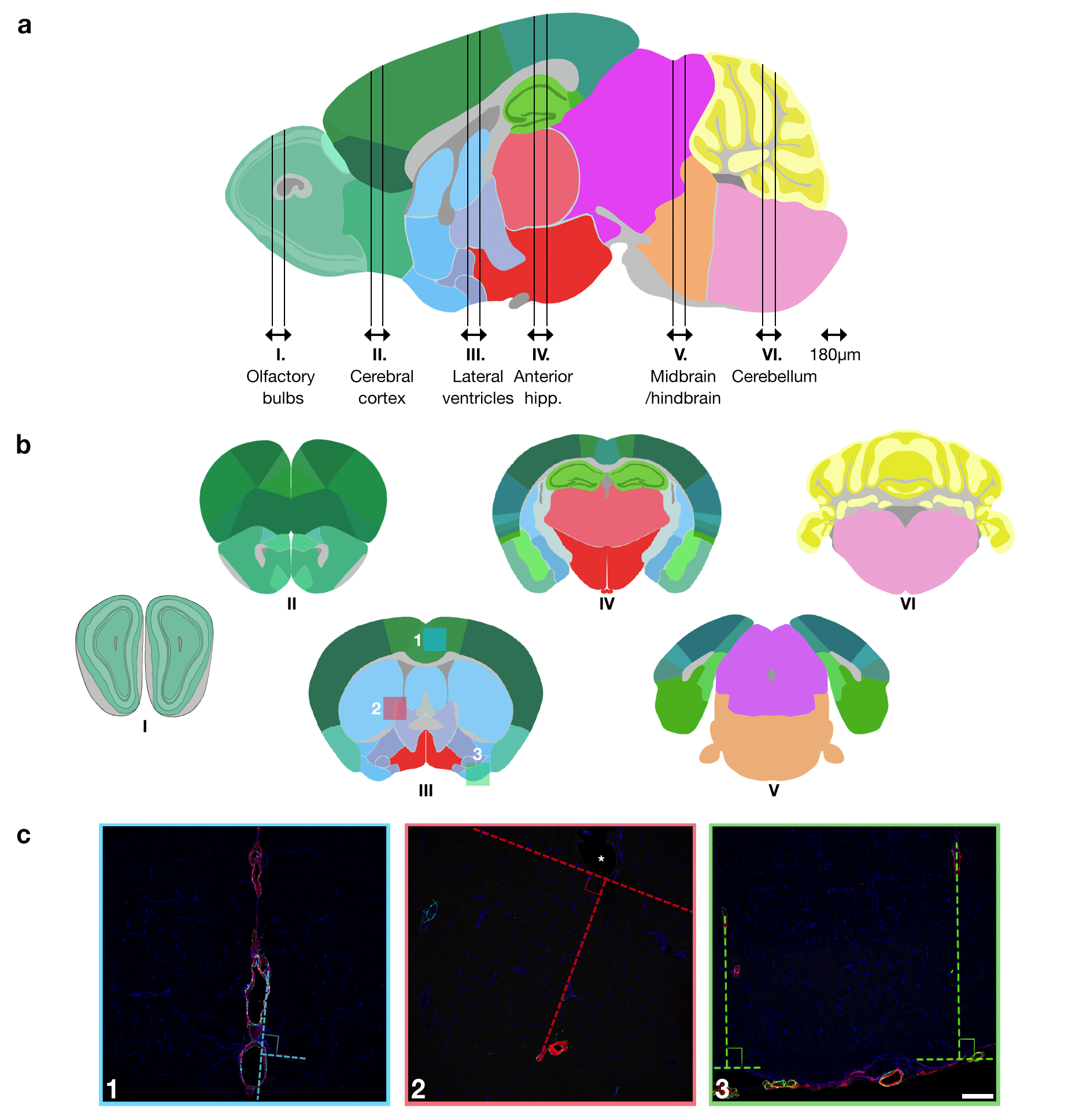

Supplement: Supplementary file 2 — Method for measuring distance of periarterial Aβ penetration into the brain. (a) Sagittal view of mouse brain illustrating the locations (I–VI) at which representative slices were taken to analyse the penetration of Aβ in the brain from the CSF. (© 2018 Allen Institute for Brain Science. Allen Mouse Brain Atlas. Available from: http://mouse.brain-map.org/static/atlas.) (A, I-VI) Sections were taken from the level of the olfactory bulbs, cerebral cortex, lateral ventricles, anterior hippocampus, midbrain/hindbrain and cerebellum. The total length of each level analysed per mouse (n = 3/group) was 180 μm (double headed arrow). (b) Coronal view of the brain levels selected for analysis; mouse brains for this study were sectioned coronally. (c) Confocal micrographs corresponding with the boxes in B, III (1-3). (c, 1) Micrograph illustrating two perpendicular lines, a tangent to the midline fissure in the cerebral and another line in the direction of the Aβ-positive vessel. (c, 2) Same quantification method applied to the Aβ-positive vessel in the caudoputamen in relation to the lateral ventricle (asterisk). (c, 3) Distance of Aβ-positive vessel measured in the striatum in relation to the base of the brain. Scale bar: 150 μm (TIFF 10671 kb) [file 401_2018_1862_MOESM2_ESM.tiff]

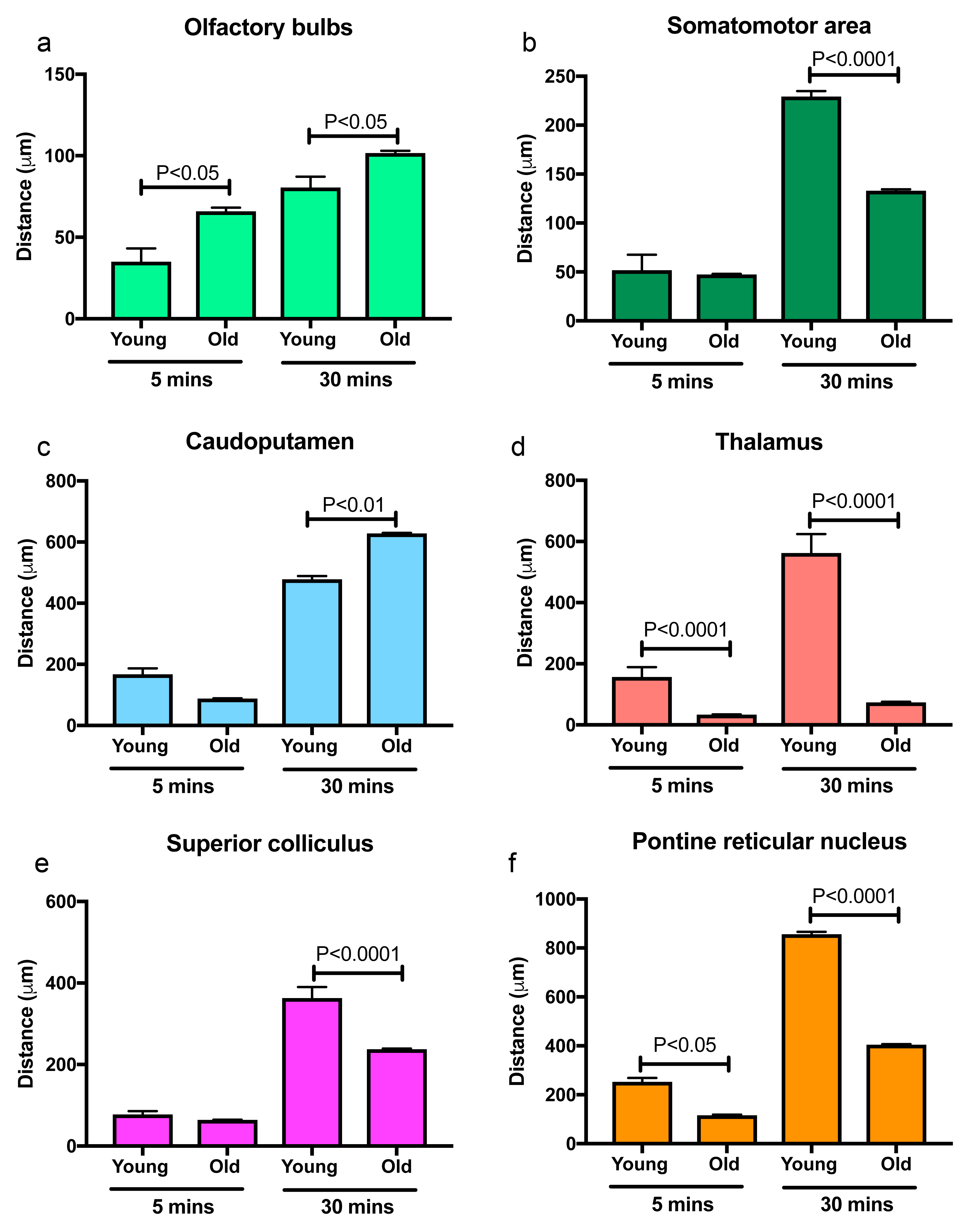

Supplement: Supplementary file 3 — Distance of periarterial Aβ penetration into the brain in young and old mice at 5 and 30 mins post injection. Bar charts of periarterial Aβ distance against time after injection into cisternal CSF (5 and 30 minutes) in the olfactory bulbs (a), somatomotor area (b), caudoputamen (c), thalamus (d), superior colliculus (e) and pontine reticular nucleus (f) of young and old mice. Values are presented as mean ± SEM of untransformed data, with p values indicated for log10-transformed data (two-way ANOVA with Sidak’s post hoc) (TIFF 553 kb) [file 401_2018_1862_MOESM3_ESM.tiff]
